# Recent Advances toward the Application of Non-Thermal Technologies in Food Processing: An Insight on the Bioaccessibility of Health-Related Constituents in Plant-Based Products

**DOI:** 10.3390/foods10071538

**Published:** 2021-07-03

**Authors:** Gloria López-Gámez, Pedro Elez-Martínez, Olga Martín-Belloso, Robert Soliva-Fortuny

**Affiliations:** Department of Food Technology, Agrotecnio CERCA Center, University of Lleida, Av. Alcalde Rovira Roure, 191, 25198 Lleida, Spain; gloria.lopez@udl.cat (G.L.-G.); pedro.elez@udl.cat (P.E.-M.); olga.martin@udl.cat (O.M.-B.)

**Keywords:** phenolic compounds, carotenoids, minerals, vitamins, bioaccessibility, pulsed electric fields, high pressure processing, ultrasounds, in vitro digestion, plant-based products

## Abstract

Fruits and vegetables are rich sources of bioactive compounds and micronutrients. Some of the most abundant are phenols and carotenoids, whose consumption contributes to preventing the occurrence of degenerative diseases. Recent research has shown the potential of non-thermal processing technologies, especially pulsed electric fields (PEF), ultrasounds (US), and high pressure processing (HPP), to trigger the accumulation of bioactive compounds through the induction of a plant stress response. Furthermore, these technologies together with high pressure homogenization (HPH) also cause microstructural changes in both vegetable tissues and plant-based beverages. These modifications could enhance carotenoids, phenolic compounds, vitamins and minerals extractability, and/or bioaccessibility, which is essential to exert their positive effects on health. Nevertheless, information explaining bioaccessibility changes after non-thermal technologies is limited. Therefore, further research on food processing strategies using non-thermal technologies offers prospects to develop plant-based products with enhanced bioaccessibility of their bioactive compounds and micronutrients. In this review, we attempt to provide updated information regarding the main effects of PEF, HPP, HPH, and US on health-related compounds bioaccessibility from different vegetable matrices and the causes underlying these changes. Additionally, we propose future research on the relationship between the bioaccessibility of bioactive compounds and micronutrients, matrix structure, and non-thermal processing.

## 1. Introduction

Consumption of bioactive compounds such as carotenoids and phenolic compounds has been related to preventing degenerative or cardiovascular diseases. Humans cannot biosynthesize such phytochemicals de novo, hence their intake from food is needed. Fruits and vegetables are excellent sources of phenolic and carotenoid compounds as well as micronutrients such as minerals and vitamins. To exert their positive effects on health, bioactive compounds must be released from the food matrix and assimilated; therefore, the interest in developing new products rich in bioactive compounds with an enhanced absorption has greatly increased. Bioaccessibility is defined as the amount of a compound that is released from the food matrix through the gastrointestinal tract and is available for absorption. Therefore, the bioaccessibility of a compound is roughly more relevant than its content within the food matrix [1]. In vitro models have been widely used for determining the bioaccessibility of bioactive compounds, given that they allow simulating in vivo conditions (pH changes, electrolytes presence, and enzyme actions) while being cost-effective, rapid, and reproducible [2]. In general, the bioaccessibility of bioactive compounds depends on their content and chemical structure, matrix properties, and interactions with other components during digestion. Food processing or the addition of adjuvants (e.g., milk or oil) have the potential to modify these characteristics and affect the bioaccessibility of bioactive compounds [3]. Thermal treatments have been used for food preservation, although in some cases product quality attributes, bioactive content, and compounds bioaccessibility are negatively affected. Recently, non-thermal technologies such as pulsed electric fields (PEF), ultrasounds (US), high pressure processing (HPP), and high pressure homogenization (HPH) have been proposed as alternatives to conventional thermal processing since health-related properties of plant-based foods can be improved. Thereby, their application to whole plant products can induce the accumulation of bioactive compounds by triggering a stress defense response. Furthermore, these technologies also modify the structure in tissues that can lead to improving the bioactive compound’s bioaccessibility. The application of PEF causes the formation of pores in cell membranes as a result of an electrical breakdown, promoting the leakage of cell content. Regarding US, its application generates shock waves that affect the structure of the cell wall and membrane. Likewise, the application of HPP induces chemical reactions and physical transformations that imply the modification of the cell wall’s integrity [4]. HPH is applied to liquid products and it consists of generating a pressure gradient between the inlet and outlet of an orifice in which pressurized fluid is passing. This causes cavitation and shear forces that will affect the food matrix structure [4]. Additionally, pulsed light (PL) and cold plasma (CP) belong to non-thermal processing technologies. Nevertheless, their effect is mainly exerted on the food surface, while the internal structure of the product is not directly affected. Therefore, we decided to discuss the non-thermal technologies that exert a strong effect on food structure because this is directly related to changes in bioaccessibility.

**Figure 1:**
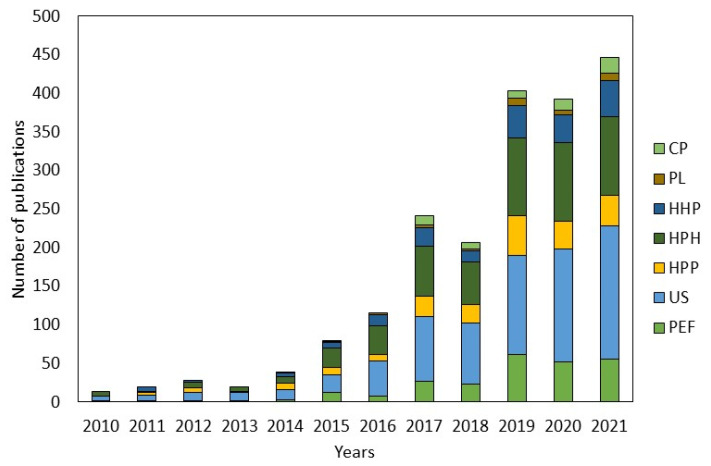
Number of publications about the effect of non-thermal processing technologies on bioaccessibility. CP: Cold plasma; PL: Pulsed light; HHP: High hydrostatic pressure; HPH: High pressure homogenization; HPP: High pressure processing; US: Ultrasounds; PEF: Pulsed electric fields.

**Figure 2:**
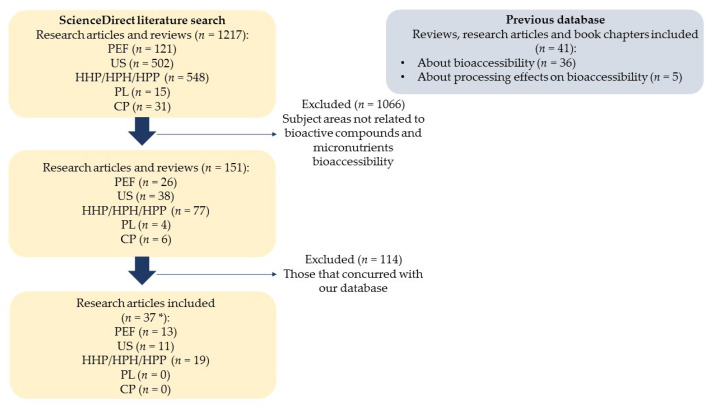
Search criteria for conduction of literature search with keywords “pulsed electric fields” AND “bioaccessibility”, “ultrasounds” AND “bioaccessibility”, “pulsed light” AND “bioaccessibility”, “cold plasma” AND “bioaccessibility”, “high pressure processing” AND “bioaccessibility”, “high hydrostatic pressure” AND “bioaccessibility”, and “high pressure homogenization” AND “bioaccessibility” and excluding results not related to bioactive compounds and micronutrients bioaccessibility. For the review, 78 research documents were referred. * *n* = 37 because 5 research articles compared in the same manuscript the effect of different processing technologies, therefore those that concurred were removed. CP: Cold plasma; PL: Pulsed light; HHP: High hydrostatic pressure; HPH: High pressure homogenization; HPP: High pressure processing; US: Ultrasounds; PEF: Pulsed electric fields.

The literature utilized for writing the “Factors Affecting Bioaccessibility of Bioactive Compounds and Micronutrients” section was based on author experience. Twelve reviews, six book chapters and eighteen research articles about the bioaccessibility of bioactive compounds and micronutrients and five reviews about the processing effect on their bioaccessibility were used. Furthermore, a systematic search was conducted to include the most recent articles about the effect of non-thermal technologies on the bioaccessibility of bioactive compounds and micronutrients. The search was carried out in the database ScienceDirect, which was selected based on their huge collection of publications, from 3800 journals and 35,000 books. Boolean operators and keywords used to search were: “pulsed electric fields” AND “bioaccessibility”, “ultrasounds” AND “bioaccessibility”, “pulsed light” AND “bioaccessibility”, and “cold plasma” AND “bioaccessibility”. Additionally, in order to perform a deep search on the high pressure processing articles, we analyzed the results of different keyword combinations: “high pressure processing” AND “bioaccessibility”, “high hydrostatic pressure” AND “bioaccessibility”, and “high pressure homogenization” AND “bioaccessibility”; after that, we eliminated those that converged. Used filters were article type “review articles”, “research articles” and years “from 2010 until 2021”. Figure 1 shows the number of publications about analyzed subjects during the last eleven years. Publications were reviewed and those that did not study the bioaccessibility of micronutrients or bioactive compounds in plant-based food were discarded. Finally, we also discarded those manuscripts that concurred with those that we already had in the database. Regarding the effect of cold plasma and pulsed light processing on bioaccessibility just four research articles were collected. That is the second reason we decided not to include them in this review (Figure 2). In the next sections, the effect of non-thermal technologies (PEF, HPP, HPH, and US) on the bioaccessibility of carotenoids, phenols, and micronutrients will be discussed by compiling updated studies to support this information.

## 2. Factors Affecting Bioaccessibility of Bioactive Compounds and Micronutrients

### 2.1. Carotenoids

Carotenoids are lipophilic pigments consisting of 40-carbon molecules and multiple conjugated double bonds [5]. Chemically, carotenoids can be divided into xanthophylls, which contain one or more oxygenated groups in their structure (e.g., lutein and zeaxanthin), and carotenes, which are unoxygenated carotenoids (e.g., lycopene and β-carotene) [6]. Carotenoids can be found as ci*s* or trans isomers due to their structure rich in conjugated doubled bonds. The long-chain carotenoids are much more prone to oxidization and isomerization, which could occur during processing and storage [6].

In order to be bioaccessible, carotenoids must be released from the food matrix during digestion. In fruit and vegetables, carotenoids are usually stored in chromoplasts, which together with the cell wall and membrane act as natural barriers for their release. Once carotenoids have reached the intestine, they must be incorporated into mixed micelles to be absorbed. Pancreatic secretions and bile salts are required to release fat-soluble compounds and aid their partition into lipid droplets (micelles) [7]. Diverse factors may interfere with their bioaccessibility: food matrix and structure, their concentration, deposition and distribution in chromoplasts, their chemical structure, or their linkages to other constituents (dietary fiber, proteins, carbohydrates, among others). In general, those compounds with a more flexible structure are easily absorbed (e.g., phytoene) [8,9]. Furthermore, Tyssandier et al. [10] suggested that less hydrophobic compounds (e.g., xanthophylls) would be easily transferred to micelles due to their location in the surface of lipid droplets, which is supported by several studies [9,11,12]. Nevertheless, carotenoid bioaccessibility is not only dependent on the food matrix or chemical structure, as it has been reported that the same product may contain carotenoids that, even though presenting a similar structure, differ in their absorption. This is the case of ζ-carotene and lycopene, which, in tomato products, are highly and poorly bioaccessible compounds, respectively; this difference in absorption could be related to their deposition form, as reported by Panozzo et al. [13] in tomato varieties or Palmero et al. [14] in tomato and carrot varieties. Carotenoids are synthesized and stored in diverse types of chromoplasts and also deposited differently (solid crystalloid, plastoglobuli, or globular-tubular) depending on the food product. Their physical state could prevent their absorption during digestion (crystalloid aggregates) such as in carrots and tomatoes or could enable an efficient release and assimilation (globular–tubular form), as in butter squash or sweet potato [12,15,16]. Additionally, cell properties (membrane thickness, size, organization) and linkages with other compounds also play an important role in this phenomenon. Jeffery et al. [15] established that a fibrous cell wall, compact cell organization and small cell size may reduce bioaccessibility as well as the presence of large amounts of dietary fiber, which impairs micelles formation, thereby blocking carotenoid absorption in the small intestine [17]. This effect is clearly observed in raw products, but some types of processing are also prone to modifying the rheological properties of liquid matrices.

In addition, some of them can be positively or negatively affected by processing technologies, cooking methods, the addition of products rich in lipids or proteins (oil, milk, among others), or ultimately a combination of these factors. Previous studies have proven that the disruption or weakening of natural barriers (cell wall, membrane, and chromoplasts) is essential for improving carotenoid bioaccessibility [14]. Therefore, food processing has become a valuable tool for this purpose, as reported by numerous authors [18].

### 2.2. Phenolic Compounds

Phenolic compounds are characterized by the presence of one or more aromatic rings, which include at least a hydroxyl group. It is a heterogeneous group that can be classified based on its structure: phenolic acids, flavonoids, coumarins, stilbenes, or lignans. Phenolic compounds are mostly linked to carbohydrates or organic acids, although some of them are also stored in vacuoles or present in the cytosol where they are synthesized [19]. They can be found in different forms in plants, such as aglycones (free phenolic acids), esters, or glycosides. As occurs with carotenoids, to be absorbed, phenolic compounds must be first released from the food matrix. Afterwards, they are either assimilated in the small intestine or further fermented in the colon if they are linked to dietary fiber.

Phenolic bioaccessibility is affected by several factors such as food matrix, chemical structure, interactions with other compounds, and food processing. Their molecular structure strongly affects their bioaccessibility. For instance, anthocyanins are very sensitive to degradation in the gastrointestinal tract but isoflavones are highly stable [3]. In addition, phenolic acids can be easily absorbed in aglycone form, but those esterified are less bioaccessible because ester bonds need to be hydrolyzed [3].

Processing generally reduces particle size, which has been associated with enhanced phenolic bioaccessibility [3]. However, it also enables the creation of new interactions between compounds characterized by the presence of hydrophobic aromatic rings and hydroxyl groups and other macromolecules such as polysaccharides (starch, cellulose, and pectin), proteins and lipids [20], which would affect bioaccessibility. Those compounds with high molecular weight or a high number of hydroxyl groups interact more with polysaccharides (H-bonds, or hydrophobic interactions) than those with low molecular weight, which hinders their bioaccessibility [21]. Molecular weight, structural flexibility, and the number of hydroxyl groups also play an important role in the formation of protein–phenol interactions, which can act as carriers during digestion. Likewise, interactions with lipids could be protective for phenolic compounds during the gastrointestinal tract, which can be positive for enhancing bioaccessibility [21]. To the best of our knowledge, the information about the effect of dietary lipids on phenolic bioaccessibility is limited. Although most phenolic compounds are hydrophilic, more apolar compounds (e.g., curcumin) can be positively affected by the presence of lipids, as mixed micelles can stabilize them [22].

Several studies have demonstrated that the disruption of cell walls and cellular compartments as well as the decrease in particle size, are essential for phenolic release and absorption, which can be achieved through processing technologies [3]. Nevertheless, information about the effect of non-thermal technologies on phenolic bioaccessibility is rather limited.

### 2.3. Minerals

Some minerals are essential for the body to perform vital functions (e.g., Ca, Fe, Mg, and Zn), therefore, their bioaccessibility is highly relevant to maintain normal metabolic functioning. After ingestion of food, most minerals are absorbed in the small intestine and transported into the bloodstream through active and passive processes [23]. Minerals’ bioaccessibility depends on the content, composition, and chemical species of each mineral, as well as the presence of promoters (e.g., organic acids) or inhibitors (e.g., phytate, oxalates, fibers) of absorption, also known as antinutrients [24]. In order to evaluate mineral bioaccessibility, their solubility is studied since it correlates well to their intestinal absorption [25]. However, some authors have reported that the dialysis method is a better indicator of bioaccessibility than the former because the presence of some constituents in the food matrix can alter their solubility [24]. Dialysis involves mineral transport through a semipermeable membrane with a fixed pore size, which reproduces gastrointestinal conditions more accurately [24].

Some processing technologies cause changes in minerals’ bioaccessibility because their application decreases antinutrient content or increases organic acid content. Organic acids can be bound to minerals and form soluble ligands, which prevent the formation of insoluble complexes with phytate. In addition, the activation of phytases could also be beneficial for mineral bioaccessibility [26].

### 2.4. Vitamins

Some vitamins can be synthesized by the human body, although certain levels of intake by diet are necessary to cover the organism demand. Vitamins can be classified into two groups depending on their solubility, which will be differently absorbed during digestion. Fat-soluble vitamins have low molecular weight and are not soluble in water (vitamins A, D, E, and K). These must be released from the food matrix and incorporated into micelles to be absorbed in the small intestine [27]. On the other hand, water-soluble vitamins (vitamin C and the B group) have one or more polar groups and are highly soluble in polar environments. They need to be released from the matrix to be absorbed in the small intestine, but contrary to fat-soluble vitamins, they are absorbed by active transport instead of being micellarized [23].

Vitamins’ bioaccessibility depends on several factors such as their chemical structure and physical state, temperature, light, pH, and oxygen, or their interaction with other compounds. For instance, sulfur or glutathione presence favors vitamin C stability, whereas fructose can exert a negative influence. Therefore, processing technologies can also influence some of these factors, and the stability and bioaccessibility of vitamins [28].

## 3. Impact of Non-Thermal Processing Technologies on Bioaccessibility

### 3.1. Carotenoids

Thermal processing has been conventionally applied in order to obtain microbiologically safe products. Some studies demonstrate that the bioaccessibility of bioactive compounds is also improved. However, high temperatures may lead to the degradation or isomerization of carotenoids while causing detrimental effects on sensory and nutritional quality attributes. Non-thermal processing technologies such as PEF, HPP, HPH, and US have been proposed as alternative processing techniques. Table 1 shows some studies that focused on the effects of processing by non-thermal technologies (PEF, HPP, HPH, US, or combinations with other technologies) in the carotenoid bioaccessibility of different food matrices.

Individual carotenoids are differently absorbed depending on intrinsic characteristics such as their molecular structure and deposition form. Generally, those carotenoids characterized by a linear shape and a high number of double bonds have more possibilities of aggregation [50], which explains the lower bioaccessibility of trans isomers. Some processing technologies are capable of modifying bioaccessibility by causing isomerization. For instance, Zhang et al. [35] reported that US (800 W; 20 min; 25 kHz) application improved carotenoid bioaccessibility by favoring the isomerization of trans to cis-lycopene in tomato juice. Conversely, Knockaert et al. [31] reported that HPP application for sterilizing tomato puree (600 MPa at 117 °C) caused the formation of compact aggregates, which negatively affected carotenoid bioaccessibility inhibiting the isomerization to cis-lycopene.

The formation of fiber networks has been reported after applying HPP and US, which has been associated with decreases in carotenoid bioaccessibility. Likewise, during digestion, pectin characteristics also affect the formation of micelles by binding bile salts or interacting with lipase [51]. These properties can be modified by applying processing technologies such as HPP. Colle et al. [33] investigated the effect of HPH (84–1327 bar) or its combination with heating (90 °C for 30 min) on the bioaccessibility of lycopene from tomato pulp. They reported that it decreased when increasing the applied pressure. In the same line, Panozzo et al. [13] found an inverse relationship between tomato pulp’s consistency (caused by the formation of fiber networks) treated by HPH (single pass at 20–100 MPa) and carotenoid bioaccessibility (lycopene, lutein, and ζ-carotene). Like other authors, they attributed this effect to the formation of a fiber network together with the low amount of lipids present in samples, which would cause the micellarization to be difficult. Nevertheless, Liu et al. [45] found that carotenoid bioaccessibility from carrot juice submitted to HPH (20–180 MPa; pass of 1, 2, or 3) increased by up to 30–50%, whereas carotenoid bioaccessibility of untreated juices was 25%. Authors delved into the relationship between pectin characteristics and carotenoid bioaccessibility and found a correlation in which lower methoxylation degree and emulsifying activity of pectin caused a higher bioaccessibility. This is because high methoxylated pectin forms a closed structure around lipid droplets that restrict lipase access and prevent lipid digestion. Besides, the high emulsifying activity contributes to encapsulate carotenoids, thus avoiding their absorption. Gence et al. [52] also reported that the presence of large amounts of pectin in citrus juices seemed to modify the size of the micelles and cause a decrease in carotenoid bioaccessibility (from 10–15% until 2.5%). On the other hand, Cano et al. [39] reported an increase in carotenoid bioaccessibility in HPP-treated (200 MPa for 6 min) persimmons. Carotenoids from untreated persimmons were not bioaccessible, whereas carotenoid bioaccessibility of those HPP-treated reached 36.9%. This effect was attributed to changes in the intermolecular interactions among the exposed hydrophobic groups of pectin during treatments.

Likewise, numerous studies have related a smaller particle size with carotenoid bioaccessibility [40,44,53]. Recently, Sentandreu et al. [34] assessed the effects of HPH (150 MPa reaching a temperature of 68 °C for 15 s) and conventional pasteurization (65 °C for 15 s, 85 °C for 15 s, and 92 °C for 30 s) on carotenoid bioaccessibility from mandarin juice. Pasteurization did not cause any remarkable change in either particle size or bioaccessibility. However, HPH increased the total carotenoid bioaccessibility 5-fold due to the decrease in particle size that allowed digestive enzymes to easily access. In a similar way, US also affects pectin properties and, consequently, carotenoid bioaccessibility. Anese et al. [37] reported that US (15, 30, and 60 min; 100 µm; 105 W/cm^2^) caused pectin de-esterification, leading to the formation of a strong network that increased the viscosity of treated tomato pulps. Additionally, they found an exponential and negative relationship between the viscosity of tomato pulps treated by US and lycopene bioaccessibility. Nevertheless, some studies have reported a positive effect in carotenoid bioaccessibility after applying US. Buniowska et al. [42] investigated the effect of applying the same specific energies (32 and 256 kJ/kg) of PEF and US in a fruit juice sweetened with *Stevia rebaudiana*. They found that total carotenoids were better absorbed after applying both PEF treatments and US (32 kJ/kg) and attributed these changes to modifications in juice rheological properties that favored the accessibility of digestive enzymes. The authors did not provide information about particle size distribution, which probably was determinant for the bioaccessibility increase. Mercado-Mercado et al. [38] also reported a 30–50% enhancement in carotenoid bioaccessibility (lutein, β-carotene, and β-cryptoxanthin) after applying US (90 W; 24 kHz; 30 min) in mango by-products, which was attributed to modifications in the structure of soluble and insoluble fibers by breaking ester bonds and hydrogen bonds, among others. Recently, de Souza Carvalho et al. [46] evaluated the influence of US (0, 0.9, 1.8, 2.7, and 3.6 kJ/cm^3^) in the carotenoid bioaccessibility of buriti juices (*Mauritia flexuosa*, Brazilian fruit). β-Carotene content and bioaccessibility increased when treatments characterized by higher ultrasound energy density were applied, doubling that of untreated juice at 3.6 kJ/cm^3^. US is likely to have promoted the rupture of cell walls and affected protein–carotenoid complexes, which increased their release before and during digestion.

Limited information is available concerning the effect of PEF on carotenoid bioaccessibility. López-Gámez et al. [30] reported that total carotenoid, α-carotene, and β-carotene bioaccessibility in PEF-treated carrots (five pulses of 3.5 kV/cm) increased by 80.2%, 59.1%, and 58%, respectively. They attributed this increment to electropermeabilization and changes in the location of carotenoids inside the cells, which were probably better released from the matrix during digestion. Some authors have studied the feasibility of using PEF on whole matrices as a pre-treatment to obtain from them a derived product with enhanced bioactive content and bioaccessibility. For instance, González-Casado et al. [31] investigated carotenoid bioaccessibility from low-fat tomato purees obtained from PEF-treated tomatoes (0.4–2 kV/cm; 5, 18, 30 pulses; 0.02–2.31 kJ/kg). The PEF-treated whole tomatoes showed increased carotenoid content, which was attributed to two different causes: (1) the triggering of carotenogenic pathways by activation of the plant’s secondary metabolism and (2) a better extractability caused by structural modifications. From the treated fruit, they produced low-fat purees (5% olive oil) with an enhanced carotenoid content, whereas the bioaccessibility of some compounds increased (Table 1). Nevertheless, some treatments decreased their bioaccessibility, which was explained by a competitive inhibition between carotenoids during micellar incorporation provoked by the elevated content initially found in the matrix. In the same line, Jayathunge et al. [33] observed an increased bioaccessibility of trans-(6.2%) and cis-(31%) lycopene immediately and 24 h, respectively, after applying PEF (4 μs of 1 kV/cm) to whole tomatoes. Thereafter, they obtained their juices and these were treated by thermal (95 °C for 20 min) and non-thermal technologies (US; PEF; US + PEF) (Table 1). US and thermal treatments decreased trans-lycopene bioaccessibility, but that of cis-lycopene was enhanced. Authors attributed the decrease to the formation of fiber networks that hinder their release during digestion given that trans isomers are more easily entrapped than cis isoforms.

Unfortunately, there are still no other studies in which PEF has been applied to whole products to improve bioaccessibility. However, PEF has been combined with heating to understand how these technologies and natural barriers affect carotenoid bioaccessibility. Bot et al. [32] studied the effect of PEF (7.6 MJ/kg; 40–45 °C), heating (7.6 MJ/kg; 85–90 °C), or its combination in tomato fractions (tissue, cell clusters, single cells, and chromoplasts). The highest bioaccessibility of β-carotene and all-trans-lycopene was observed in chromoplast fraction, which was attributed to the absence of cell wall polysaccharides that impair the action of digestive enzymes. However, bioaccessibility decreased after applying PEF, which was associated with the formation of carotenoid–protein complexes [54], given that PEF can induce modifications in protein conformation [55]. In tomato tissues, all-trans-lycopene bioaccessibility decreased after heating, which is in accordance with results reported by Jayathunge et al. [33], whereas β-carotene was not affected, probably due to their different chemical structure [56]. In cell clusters or single cells, bioaccessibility was not affected by any treatments, which seems to indicate that the effect of PEF strongly depends on the complexity of structural barriers.

Regarding the effect of adjuvant addition, several studies have demonstrated that oil incorporation during processing or digestion is essential to enhance carotenoid bioaccessibility because it promotes their micellarization. It has been reported that lipids with a high unsaturation degree and a long-chain length of fatty acids (e.g., olive oil) are better enhancers of bioaccessibility than those of short or middle length (e.g., coconut oil). Besides, both the type and quantity of added oil are crucial for enhancing bioaccessibility, which is highly related to the lipid hydrolysis mechanism [57]. Monoacylglycerols and free fatty acids contribute to the formation of mixed micelles that encapsulate carotenoids when lipid load is low. However, when lipid load is higher, the hydrolysis of triglycerides is incomplete, which leads to the formation of a lipid phase that entraps carotenoids, avoiding their micellarization. The effect of applying non-thermal technologies to food products with added oil on carotenoid bioaccessibility has been reported in several studies. Anese et al. [36] evaluated the addition of sunflower oil (2.5, 5, and 10%) and US treatment (30 min; 24 kHz; 100 µm; 1462 J/cm^3^) on lycopene bioaccessibility of tomato pulp. Results showed that an increase in the percentage of oil caused a decreased bioaccessibility and US treatment did not affect it. Authors reported a better carotenoid release from cells, but the formation of a fiber network due to US application could entrap carotenoids and hinder their micellarization. The effect of HPH (20 MPa) and the addition of olive oil in tomato and carrot-derived suspensions was evaluated by Moelants et al. [40]. Authors reported that β-carotene bioaccessibility was enhanced when a 2% oil-in-water emulsion instead of 2% olive oil was added. By emulsifying, the oil surface area increases, and the transfer of hydrophobic carotenoids to the lipophilic phase can be enhanced. Furthermore, bioaccessibility clearly increased when cells were broken and particle size was lower than 125 μm, which is approximately the carrot cell size. The authors found that emulsion addition led to a greater increase in carotenoid uptake into the micellar phase, followed by just the olive oil. Similar results on particle size reduction in HPH-treated (10, 50, or 100 MPa for 1 cycle) carrot purees (with or without oil) were reported by Knockaert et al. [44]. β-Carotene bioaccessibility was promoted when applied pressure was increased, although oil addition did not cause a further increase. Those HPH-treated purees had similar carotenoids bioaccessibility than those untreated. Under high pressure, the acyl chains of the phospholipids are straightened and packed more tightly, reducing membrane fluidity, and avoiding carotenoids release. In addition, oil can crystallize under certain pressure-temperature conditions which might hinder the solubilization of β-carotene in the oil.

### 3.2. Phenolic Compounds

Some authors have investigated phenolic bioaccessibility after applying non-thermal technologies to whole fruit and vegetables. Table 2 summarizes the main findings regarding their impact on phenolic bioaccessibility. Ribas-Agustí et al. [58] evaluated phenolic bioaccessibility of PEF-treated apples (0.01, 1.8, and 7.3 kJ/kg). 

Generally, more intense treatments caused greater bioaccessibility likely due to cell disruption and induced structural changes, given that an inverse correlation between some individual compound’s (5-caffeoylquinic acid, epicatechin, and phloretin xyloglucoside) bioaccessibility and apple toughness was found. Some of them were easily released due to microstructural changes caused by electropermeabilization, but others were independent of matrix structure (p-coumaroylquinic acid, phloretin glucoside, and quercetin derivatives). In addition, phenolic profile and content in undigested apples varied after storage and PEF treatments, which was also important for bioaccessibility. López-Gámez et al. [30] reported an increase of 53% in total phenolic bioaccessibility when carrots were treated by PEF (five pulses of 3.5 kV/cm), although some individual compounds were less bioaccessible (e.g., ferulic acid or 3-caffeoylquinic acid). The authors explained that electropermeabilization can favor phenolic release from the matrix, enhancing their bioaccessibility. However, some compounds with high molecular weight or high number of hydroxyl groups are also able to be entrapped by dietary fibers or form complexes that hinder their dialysis. Likewise, Lafarga et al. [61] investigated the effect of US (250 W for 20 min; 40 kHz; 4 °C) on the total phenolic content and bioaccessibility of different vegetables. Phenolic content increased in undigested matrices but bioaccessibility was only higher in lettuce and green pepper. The authors attributed these changes to a better release caused by acoustic cavitation phenomena and cell wall disruption, although microstructure images and firmness data were not provided to confirm this hypothesis. In the same way, Zudaire et al. [60] also studied the effect of digestion and US (40 kHz; 250 W at 0, 10, 25, 45 min) processing on the total phenolic content of calçots, a type of green onion. After 10 min of treatment, total phenolic content in digesta was lower than that of untreated calçots, which was attributed to microchannels created by US. This enabled phenolic compounds to be easily released and to be more exposed to degradation by pH during digestion.

Some authors have studied the effect of non-thermal technologies on phenols bioaccessibility in liquid matrices. Ribeiro et al. [62] evaluated phenolic and anthocyanin bioaccessibility of juçara smoothies treated by US (220 W for 7 min). No differences in phenolic bioaccessibility were found in untreated smoothies, whereas anthocyanins were more bioaccessible after US treatment. It is likely that cavitation bubbles cause the breakage of cells and phenolic release, but the higher particle size and the increase in viscosity caused by US impairs their correct absorption. On the other hand, anthocyanins are sensitive to changes in pH. Therefore, they were probably protected from degradation during digestion through the formation of the same aggregates that impaired phenolic absorption. The influence of US (0, 0.9, 1.8, 2.7, and 3.6 kJ/cm^3^) in the anthocyanin bioaccessibility of açai juices was evaluated by de Souza Carvalho et al. [46]. The authors reported increases in both monomeric anthocyanin content and bioaccessibility in juices treated with the highest energy density, which was attributed to cell wall rupture and their increased release.

Buniowska et al. [42] evaluated phenolic bioaccessibility of a fruit juice composed of mango and papaya, and sweetened with *Stevia rebaudiana,* after submitting them to PEF or US treatments (32 and 256 kJ/kg). Those juices treated by PEF (256 kJ/kg) and US (32 and 256 kJ/kg) showed higher bioaccessibility than those untreated, which was attributed to their better release from the food matrix. In addition, Rodríguez-Roque et al. [63] evaluated phenol bioaccessibility from a fruit beverage after being submitted to HPP (400 MPa for 5 min), PEF (35 kV/cm for 1800 μs), or thermal treatment (90 °C for 1 min). Total phenol bioaccessibility was enhanced after applying any one of the compared processing technologies. Nevertheless, individual compounds were differently affected depending on their chemical structure. PEF and HPP improved bioaccessibility of several phenolic substances, whereas the lowest increases were obtained after thermal treatments. The authors proposed that processing caused changes in the phenol structure (hydroxylation, methylation, glycosylation, among others) that enabled their absorption. Also, their degradation or conjugation may occur during digestion, causing a decrease in bioaccessibility. Recently, Rodríguez-Roque et al. [64] investigated the effect of applying PEF (35 kV/cm for 1800 µs) and HPP (400 MPa for 5 min) to a fruit and soymilk-based beverage. They observed that this type of processing improved isoflavone bioaccessibility compared to those untreated or thermally treated (90 °C for 1 min). The authors attributed this effect to the disruption of cell membranes in which protein–isoflavone complexes are embedded.

Some studies on phenolic bioaccessibility after non-thermal processing, and the addition of different types of milk have been performed. He et al. [59] and Quan et al. [65] evaluated the effect of HPH (250 MPa for 10 min), thermal treatments (80 °C for 30 min or 90 °C for 30 s), and the type of milk added (whole, skimmed or soymilk) on polyphenols of juices obtained from different matrices (apple, grape, orange, pomelo, and kiwi). In grape and orange juices, bovine milk and thermal treatments were more appropriate than soymilk and HPH to enhance bioaccessibility. However, in the case of apple and pomelo juices, decreased or similar bioaccessibility values were observed after both treatments and types of milk. On the other hand, kiwi juice had a greater bioaccessibility regardless of the type of milk added (21.6–37.7%), although processing did not improve it. Obtained results depended on the matrix and their specific compounds. Some of them are prone to interact with milk proteins, leading to the formation of complexes and insoluble aggregates that, consequently, decrease bioaccessibility. It would be interesting to evaluate the viscosity of juices or fiber content to determine if this is a factor to be considered. Similar results were reported by Rodríguez-Roque et al. [63], who also investigated the effect of adding milk (whole, skimmed, or soymilk) to fruit beverages. The highest phenolic bioaccessibility was obtained in juices that contained whole milk, but those with skimmed or soymilk showed lower bioaccessibility. Whole, skimmed, and soymilk present a high amount of proteins, which could favor the formation of complexes with phenolic substances and their precipitation, whereas lipid content in whole milk did not favor that association.

According to Alminger et al. [66] several changes in the structure (hydroxylation, methylation, glycosylation) of these compounds occur during digestion, as well as the formation of other derivatives. Likewise, it is important to highlight that a large proportion of polyphenols (e.g., ferulic or caffeic acid) are associated with dietary fiber, which make their absorption difficult [19]. Hence, they cannot be extracted with common solvents (e.g., methanol) in undigested foods, but they can be released during digestion due to pH conditions [67]. This fact leads to the underestimating of phenolic content, which directly affects bioaccessibility calculation. Some alternatives to correctly quantify phenolic content have been carried out by Mercado-Mercado et al. [68], who determined the amount of free phenolic compounds as well as those associated with dietary fiber.

### 3.3. Minerals

Limited information has been reported about the effects of non-thermal processing technologies on minerals bioaccessibility. Most of the studies investigate the effect of HPP on legumes or cereals (Table 3). The reported results demonstrate that bioaccessibility can be enhanced due to the formation of non-covalent bonds and microstructural changes that favor the accessibility of digestive enzymes [69]. In addition, antinutritional factors (i.e., phytates and fibers) can be reduced, which will enhance mineral bioaccessibility [23]. For instance, the application of HPP (500 MPa for 10 min) to algarroba seeds led to triple calcium and iron bioaccessibility and quintuple that of zinc, which could be related to microstructural modifications [70]. On the other hand, it has been reported that HPP can also reduce calcium bioaccessibility in HPP-treated apples (500 MPa for 2–10 min) whereas that of iron and zinc were differently affected depending on the intensity of the applied treatment. Their dialysability may be enhanced by cell wall disruption and the formation of low molecular weight complexes [25]. In another study carried out by Xia et al. [69], authors reported that copper bioaccessibility was increased by 2.87–23.06% in HPP-treated brown rice (300 and 500 MPa for 10 min), but bioaccessibility of iron was decreased after treatments (100, 300, and 500 MPa for 10 min).

The bioaccessibility of minerals in liquid products has not been extensively studied. Cilla et al. [71] reported increases in bioaccessibility and bioavailability of phosphorous and calcium in HPP-treated (400 MPa for 5 min) milk-based fruit beverages compared to those thermally treated. However, no general recommendations can be given for improving mineral bioaccessibility under HPP processing. This has, though, been suggested as an alternative to thermal treatments, since enhanced bioaccessibility and cell uptake have been reported.

### 3.4. Vitamins

The impact of non-thermal processing on vitamin bioaccessibility has been scarcely studied and most of the studies regarding this topic are focused on vitamin C from beverages (Table 3). Vitamin C is extremely sensitive to temperature, therefore, if processing intensity is too high and the vitamin is exposed to conditions that alter their stability, it probably would not be detected after in vitro digestion, as Buniowska et al. [42] have reported in PEF- and US-treated (32 and 256 kJ/kg) fruit beverages. On the other hand, it has been reported that the application of HPP processing (400 MPa for 5 min) to milk-based beverages can lead to increased viscosity and hinder vitamin C from being absorbed due to protein aggregates formation [47]. Regarding PEF (35 kV/cm for 750 µs) effect on vitamin C absorption, Sánchez-Moreno et al. [72,73] demonstrated that it was similarly absorbed after consuming untreated gazpacho (typical Spanish vegetable beverage) and orange juice after submitting them to PEF. Likewise, Rodríguez-Roque et al. [63] also reported that neither HPP (400 MPa for 5 min) nor PEF processing (35 kV/cm for 1800 μs) caused changes in vitamin C bioaccessibility of fruit-based beverages with added milk.

The impact of applying non-thermal technologies on vitamin bioaccessibility of whole products is still under exploration. However, promising results have been obtained by Fonteles et al. [74], who observed an increased vitamin C bioaccessibility in cashew apple bagasse puree treated by US (500 W for 2, 6, or 10 min). This was attributed to cell disruption and their better release during digestion, although more studies are necessary to understand such changes.

**Table 3:** Micronutrients content, bioaccessibility, and structure of selected plant foods as affected by non-thermal processing.

Food Matrix	Processing Conditions	Structure	Micronutrients Content	Bioaccessibility Increase	Bioaccessibility Decrease	References
Apple	High pressure processing (HPP) (500 MPa for 2, 4, 8 and 10 min)	No information provided about structure	↑ Calcium (2–8 min) ↓ Zinc (2, 4 min) ↑ Zinc (10 min)	Iron (8 min) Zinc (8, 10 min)	Calcium (all treatments) Iron (4 min) Zinc (2, 4 min)	[25]
Brown rice	HPP (100, 300, 500 MPa for 10 min)	Surface non-uniform, with cavities	↓ Iron (100, 500 MPa) ↓ Zinc (100–500 MPa) ↓ Copper (100, 300 MPa)	No increases	Iron	[69]
Milk-based fruit beverages	HPP (400 MPa for 5 min)	No information provided about structure	↓ Calcium	No increases	No decreases	[71]
Mango and papaya juice sweetened with *Stevia rebaudiana*	Pulsed electric fields (PEF) (32 and 256 kJ/kg) Ultrasounds (US) (32 and 256 kJ/kg)	No information provided about structure	↓ Vitamin C	No bioaccessible	No bioaccessible	[42]
Fruit juice milk-based beverage	HPP (400 MPa for 5 min)	No information provided about structure	↓ α-tocopherol (HPP + whole and skimmed milk), γ- tocopherol (HPP + whole milk) ↑ α-tocopherol, γ- tocopherol, δ-tocopherol (HPP + soymilk) ↓ Vitamin C (HPP + skimmed milk and soymilk)	No increases	α-tocopherol, γ- tocopherol, δ-tocopherol (HPP + soymilk) Vitamin C (HPP + whole milk or soymilk)	[47]
Gazpacho (vegetable soup)	PEF (35 kV/cm for 750 µs)	No information provided about structure	No changes	No increases	No decreases	[73]
Orange juice	PEF (35 kV/cm for 750 µs)	No information provided about structure	No changes	No increases	No decreases	[72]
Fruit juice-based beverage mixed with soymilk	PEF (35 kV/cm for 1800 μs) HPP (400 MPa for 5 min)	No information provided about structure	↓ Vitamin C (PEF and HPP)	Vitamin C (HPP)	No decreases	[63]
Cashew apple bagasse puree	US (500 W for 2, 6, 10 min)	Microchannels and cell disruption	↑ Vitamin C	Vitamin C	No decreases	[74]

PEF: Pulsed electric fields; US: Ultrasounds; HPP: High pressure processing; HPH: High pressure homogenization.

## 4. Concluding Remarks and Further Research

PEF, HPP, HPH, and US can cause permeability changes in cell membranes, which are directly connected to microstructural changes in whole matrices and particle size reduction in liquid matrices. Generally, this facilitates the release of carotenoids and phenolic compounds, which improves their bioaccessibility. On the other hand, particle size reduction can also facilitate the formation of a strong fiber network due to more interactions between fragments of cells, which increase the viscosity of the product (juices and purees) and entrap carotenoids avoiding their correct absorption. Little information is available about the effect of viscosity and pectin on phenolic bioaccessibility, but it has been suggested that phenol–fiber interactions play an important role.

The presence of oils in pulps or purees is beneficial to the enhancement of carotenoid bioaccessibility, whereas its effect is still unknown for phenol bioaccessibility. Nonetheless, it has been reported that the combination between PEF or HPP and the presence of lipids could increase their bioaccessibility in liquid matrices. Therefore, the effect of processing on bioaccessibility would depend on the balance between compounds degraded or modified during processing and/or digestion and those that are protected by the matrix.

Structural properties of matrices are one of the most important factors determining bioactive compound bioaccessibility. Hence, further studies about the effect of these technologies on viscosity, fiber, particle size, pectin properties, and microstructural characteristics would be necessary to develop non-thermal strategies to enhance bioactive compound bioaccessibility and to understand the main causes of these changes. Finally, future research should also focus on shelf-life, quality-related enzyme activities, and consumer’s acceptance, given that processing may alter the typical flavor of the final product, their quality attributes, or their microbiological stability during storage.

## Figures and Tables

**Table 1:** Carotenoid content, bioaccessibility, and structure of selected plant foods as affected by non-thermal processing.

Food Matrix	Processing Conditions		Structure	Carotenoid Content	Bioaccessibility Increase	Bioaccessibility Decrease	References
Carrot	Pulsed electric fields (PEF) (0.9 and 191 kJ/kg) in water PEF (0.9 and 191 kJ/kg) in 300 ppm CaCl_2_ PEF + Blanching (B) (100 °C for 5 min)		PEF (191 kJ/kg in water or in 300 ppm CaCl_2_): Decrease hardness PEF (191 kJ/kg in CaCl_2_ + B): Increase hardness	No changes in β-carotene content	No changes	No changes	[29]
Carrot	PEF (five pulses of 3.5 kV/cm)		↓ Firmness Degradation of cell walls Changes in carotenoid location	No changes in carotenoid content	Total carotenoids, α-carotene, β-carotene	No decreases	[30]
Tomato puree (5% olive oil)	PEF (0.02–2.31 kJ/kg) applied to whole tomato		↓ Firmness (0.06–2.31 kJ/kg)	↑ Total (0.06–2.31 kJ/kg) ↑ β-carotene (0.06–0.38 kJ/kg; 1.38–2.31 kJ/kg) ↑ Lycopene (0.14–2.31 kJ/kg) ↑ Lutein (0.14, 0.5–2.31 kJ/kg) ↑ Phytofluene, phytoene, δ-carotene ↑ ɣ-carotene (0.09, 0.14, 2.31 kJ/kg)	Total (0.38 kJ/kg), β-carotene (0.38 kJ/kg), lycopene (0.09–0.38 kJ/kg; 1.38–2.31 kJ/kg), lutein (0.09–0.38 kJ/kg), ɣ-carotene (0.09–0.38; 0.83–2.31 kJ/kg)	Total (0.02 and 0.5 kJ/kg), β-carotene (0.02 and 0.06 kJ/kg), lycopene (0.02 and 0.06 kJ/kg), lutein (0.06 and 0.5 kJ/kg), phytofluene (0.02, 0.06, 0.14, 0.5, 0.83, 1.38 and 2.31 kJ/kg), phytoene, δ-carotene (0.02–0.06 kJ/kg; 0.5–2.31 kJ/kg)	[31]
Tomato fractions	PEF (7.6 MJ/kg; 40–45 °C) PEF + Heat (H) (7.6 MJ/kg; 85–90 °C)	Tissue	PEF + H: cell detachment	No changes	No increases	all-trans-lycopene (H; PEF + H)	[32]
		Cell clusters	Cell membranes damaged	↓ β-carotene (PEF; H)		No changes	
		Single cells	Cell membranes damaged	↓ β-carotene (PEF; H)		No changes	
		Chromoplasts	No differences	↓ β-carotene (PEF; H; PEF + H) and all-trans-lycopene (PEF; PEF + H)		all-trans-lycopene and β-carotene (PEF; PEF + H)	
Tomato	PEF (1 kV/cm for 0, 4, 80 or 320 μs) Storage: 0 h, 24 h or 48 h		Irregular cell wall structure by increasing holding time treatment	↑ Total lycopene (all treatments) ↑ All-trans-lycopene (80 and 320 μs) ↑ Cis-lycopene (all treatments excepting 4 μs at 0 h)	Total lycopene (4 μs at 24 h) All-trans-lycopene (4 μs at 0 h) Cis-lycopene (all treatments at 24 h and 320 μs at 0 h)	Total lycopene (80 μs; 320 μs at 24 and 48 h) All-trans-lycopene (80 and 320 μs at 0 and 24 h and 320 μs at 48 h) Cis-lycopene (all treatments at 48 h and 4 μs at 0 h)	[33]
Tomato juice	PEF (1 kV/cm for 4 μs) B (90 °C for 2 min) PEF + B PEF + B + PEF2 (35 kV/cm for 1500 μs) PEF + B + Ultrasounds (US) (20 kHz; 20% amplitude; 7 min) PEF + B + US + PEF2		No information provided about structure	↑ Total lycopene (all treatments) ↑ All-trans-lycopene (PEF, PEF + B + US, PEF + B + PEF2) ↑ Cis-lycopene (PEF, PEF + B + PEF2) ↓ Cis-lycopene (PEF + B + US, PEF + B + US + PEF2)	Cis-lycopene (all treatments) Trans-lycopene (PEF + B + PEF2, PEF + B + US + PEF2)	Trans-lycopene (B, B + US)	[33]
Mandarin juices	High pressure homogenization (HPH) (150 MPa reaching 68 °C for 15 s)		Cell rupture and ↓ particle size	↓ Total carotenoids and individual (lutein, zeaxanthin, zeinoxanthin, β-carotene, α-carotene, β-cryptoxanthin, phytofluene, phytoene, cis-violaxanthin isomers, 9-cis- violaxanthin + cis-antheraxanthin isomers, cis-anteraxanthin isomers, luteoxanthin isomers, mutatoxanthin isomers)	Total carotenoids and individual (lutein, zeaxanthin, zeinoxanthin, β-carotene, α-carotene, β-cryptoxanthin, phytofluene, phytoene, cis-violaxanthin isomers, 9-cis-violaxanthin + cis-antheraxanthin isomers, cis- anteraxanthin isomers, luteoxanthin isomers, mutatoxanthin isomers)	No decreases	[34]
Tomato juice	HPH (200, 300, 400, and 500 bar) (2 cycles of 15 min)		↓ particle size by increasing pressure	↑ All-trans-lycopene (200 bar) ↓ All-trans-lycopene (300–500 bar) ↓ 5-cis-lycopene (400 and 500 bar) ↑ 5-cis-lycopene (200 and 300 bar) ↓ 9-cis-lycopene (all treatments) ↑ 13-cis-lycopene (300 and 400 bar) ↓ 13-cis-lycopene (200 and 500 bar) ↑ β-carotene (200 bar)	All-trans-lycopene and isomers (500 bar)	All-trans-lycopene and total lycopene (200 bar)	[35]
Tomato juice	US (25 Hz; 200 W, 400 W, 600 W and 800 W for 20 min)		Similar increase in particle size in all treatments	↑ All-trans-lycopene (200 and 400 W) ↓ All-trans-lycopene (600 W) ↓ 5-cis-lycopene (all treatments) ↑ 9-cis-lycopene (200, 400, 800 W) ↑ 13-cis-lycopene, ζ-carotene (all treatments)	All-trans-lycopene and isomers (800 W)	All carotenoids (400 W)	[35]
Commercial pasteurized tomato pulp (with or without added sunflower oil)	US (30 min; 24 kHz; 100 μm; 71 W; 1462 J/cm^3^)		US-treated samples showed broken cells with lycopene distributed in the matrix	No changes in lycopene content	Lycopene (in US-treated samples with 5% of oil)	No decreases in lycopene (in US-treated samples with 0, 2.5 and 10% of oil)	[36]
Commercial pasteurized tomato pulp	US (15, 30 and 60 min; 100 μm; 105 W/cm^2^)		Loss of cell integrity when increasing time of treatments. No intact cells after 60 min. Decrease in pectin esterification degree and increase in viscosity	No changes in lycopene content	No increases	Lycopene bioaccessibility decreases by increasing processing time	[37]
Mango by-products (peel and paste)	US (30 min; 30% of amplitude; 9 W/mL)		No information available about structural characteristics	↑ β-cryptoxanthin (in peel and paste), lutein and β-carotene (both ↓ in paste and ↑ in peel) Content was determined in gastric phase	Total carotenoids, β-cryptoxanthin, lutein and β-carotene (in peel and paste)	No decreases	[38]
Astringent persimmon	High pressure processing (HPP) (200 MPa for 6 min)		No information provided about structure	↓ Total carotenoids and xanthophyll esters, lycopene, (all-trans)-lutein 3-O-laurate-3′-O- Myristate, -β-cryptoxanthin myristate, -antheraxanthin myristate-Palmitate, -zeaxanthin myristate, -antheraxanthin 3-O-Palmitate, -lutein 3-O-palmitate, -lutein dimyristate, -antheraxanthin laurate-myristate, -α-carotene, -α-cryptoxanthin, -violaxanthin, -antheraxanthin, -neoxanthin dibutyrate, -violaxanthin palmitate, -violaxanthin laurate, 9-cis neoxanthin dibutyrate and 9-cis-β-carotene ↑ (all-trans)-zeaxanthin, -β-cryptoxanthin, -β-carotene and 9-cis-α-carotene	(All-trans)-anteraxanthin, -lutein, -zeaxantin, -β-cryptoxanthin, (all-trans and 13-cis) -α-, -β-carotene, (all-trans)-violaxanthin laurate, (all-trans)-zeaxanthin palmitate, (all-trans)-β-cryptoxanthin laurate, (all-trans)-lutein 3-O-palmitate, (all-trans)-zeaxanthin myristate, (all-trans)-antheraxanthin myristate-palmitate, (all-trans)-β-cryptoxanthin myristate, (all-trans)-β-cryptoxanthin dipalmitate and lycopene	No decreases	[39]
Carrot and tomato purees	HPH at 20 MPa Blended carrot/tomato purees Homogenized carrot/tomato purees During digestion: no addition of oil, addition of olive oil (2%) or oil emulsion (2%)		Different suspensions with particle size were prepared through wet sieving technique. The cell wall of particles smaller than 125 μm was damaged.	No information provided about carotenoid content before digestion	Carrot puree without oil: All-trans-β-carotene (≤125 μm) Carrot puree with 2% oil: All-trans-β-carotene (≤125 μm) Carrot puree with 2% oil emulsion: All-trans-β-carotene (≤125 μm) Tomato puree 2% oil emulsion: all-trans-lycopene (<40 μm HPH)	No decreases	[40]
Tomato puree (5% olive oil)	High pressure pasteurization (HP-P) (HPP 450 MPa for 15 min and 20 °C and 600 MPa for 20 min and 45 °C) High pressure sterilization (HP-S) (121.1 °C for 1.5 min and 117 °C for 3 min at 600 MPa)		Particle size was the same among treatments	↑ 13-cis-lycopene (HP-S), 9-cis-lycopene (HP-S, 3 min), 5-cis-lycopene (HP-P, HP-S, 3 min) ↓ lycopene, all-trans-lycopene, (HP-S)	No increases	Lycopene in HP-S	[41]
Mango and papaya juice sweetened with *Stevia rebaudiana*	PEF (32 and 256 kJ/kg) US (32 and 256 kJ/kg)		No information provided about structure	PEF (32 kJ/kg): ↑ Total carotenoids US (32 and 256 kJ/kg): ↓ Total carotenoids	PEF (32 and 256 kJ/kg) and US (32 kJ/kg): Total carotenoids	No decreases	[42]
Tomato pulp	HPH (84–1327 bar) HPH (220, 521, 1135 bar) + H (30 min, 90 °C)		↑ Homogenization pressure resulted in the breakdown of the tomato cell aggregate structures and volumetric percentage of the small particles increased ↑ Strength of the fiber network	No changes	No increases	Decrease in lycopene by increasing pressure up to 479 MPa. After that, it remained constant	[43]
Carrot puree without oil and adding 5% olive oil	HPH (10, 50 or 100 MPa for 1 cycle) HPH (100 MPa) + HP-P (20 min at 600 MPa and 45 °C)		↓ Particle size by increasing pressure	Carrot puree (HPH): ↑ 13-cis- β-carotene Carrot puree (5% olive oil) (HPH): No changes Carrot puree (HPH + HP-P): ↓ All-trans- β-Carotene and total β-Carotene Carrot puree (5% olive oil) (HPH + HP-P): ↑ 9-cis- β -Carotene and total β-Carotene	Carrot puree and puree with added oil (HPH): β-carotene (50 MPa and 100 MPa) Carrot puree (HPH + HP-P): No changes compared to untreated, but higher bioaccessibility than just HPH treated purees	No decreases	[44]
Tomato pulps (red, orange, and yellow)	HPH (single pass at 20, 50 and 100 MPa)		Consistency increase by increasing pressure. ↓ particle dimensions Single cells and broken material (20 MPa) Cell fragments (50 MPa) Complete breakage of cells (100 MPa)	Red: Lycopene and lutein decrease by increasing pressure Orange: ↓ ζ-carotene by increasing pressure Yellow: ↓ Lutein	No increases	All carotenoids decreased in all treatments	[13]
Carrot juice	HPH (20 MPa, 60 MPa, 100 MPa, 150 MPa and 180 MPa) (fixed 1 pass at 25 °C) Pass of 1, 2 and 3 (fixed 60 MPa at 25 °C) Inlet temperature of 25 °C, 50 °C and 70 °C (fixed 60 MPa and 1 pass)		No information provided about structure	↑ Total carotenoids (180 MPa)	Total carotenoids after each pressure treatment Total carotenoids after 3 passes Total carotenoids at 50 and 70 °C	No decreases	[45]
Buriti juice	US (0, 0.9, 1.8, 2.7 and 3.6 kJ/cm^3^)		No information provided about structure	↑ β-carotene after all treatments	β-carotene after all treatments	No decreases	[46]
Fruit juice milk-based beverage	HPP (400 MPa for 5 min)		No information provided about structure	↑ Total carotenoids, neoxanthin, 9-cis-violaxanthin (HPP + whole milk), zeaxanthin, lutein (HPP + whole milk or skimmed milk), ↓ Total carotenoids (HPP + soymilk) Zeaxanthin (HPP + soymilk), lutein	Total carotenoids (HPP + soymilk) neoxanthin, 9-cis-violaxanthin (HPP + whole milk or soymilk) zeaxanthin, lutein (HPP + soymilk)	Total carotenoids (HPP + whole milk) Zeaxanthin, lutein (HPP + whole milk or skimmed milk)	[47]
Tomato juice and kale-based juice	PEF (35 kV/cm for 1000 µs) HPP (500 MPa for 3 min)		No information provided about structure	↓ β-carotene and lutein (PEF-treated kale-based juice)	Lycopene (PEF-treated tomato juice)	β-carotene (PEF-treated tomato juice)	[48]
Orange juice	HPH (150 MPa reaching 68 °C for 15 s)		No information provided about structure	↓ Total carotenoids,antheraxanthin, violaxanthin, luteoxanthin, zeaxanthin, antheraxanthin, β-cryptoxanthin, α-carotene, β-carotene, phytoene	Lutein, zeaxanthin, zeinoxanthin, β-cryptoxanthin, α-carotene, β-carotene, phytoene, phytofluene, violaxanthin, antheraxanthin, luteoxanthin, mutatoxanthin	No decreases	[49]

PEF: Pulsed electric fields; US: Ultrasounds; HPP: High pressure processing; HPH: High pressure homogenization; B: Blanching; H: Heat; HP-P: High pressure pasteurization; HP-S: High pressure sterilization.

**Table 2:** Phenolic content, bioaccessibility, and structure of selected plant foods as affected by non-thermal processes.

Food Matrix	Processing Conditions		Structure	Phenolic Content	Bioaccessibility Increase	Bioaccessibility Decrease	References
Mango and papaya juice sweetened with *Stevia rebaudiana*	Pulsed electric fields (PEF) (32 and 256 kJ/kg) Ultrasounds (US) (32 and 256 kJ/kg)		No information provided about structure	PEF (32 kJ/kg): ↑ Total phenolic content US (32 kJ/kg): ↓ Total anthocyanins	PEF (256 kJ/kg) and US (32 and 256 kJ/kg): Total phenolic content PEF (256 kJ/kg): Total anthocyanins	No decreases	[42]
Apple, grape, and orange juices	High pressure homogenization (HPH) (250 MPa for 10 min)		No information provided about structure	Apple juice: ↓ Total phenolic content, chlorogenic acid, phloridzin, epigallocatechin-3-gallate (EGCG) and hesperidin Grape juice: ↑ Total phenolic content, caffeoyl-trataric acid, proanthocyanidin; ↓ epicatechin protocatechuic-glucoside Orange juice: ↑ Total phenolic content, naringin, caffeoyl glucoside, hesperetin- rutinoside, naringenin-trisaccharide, luteolin-rutinoside; ↓ quercetin- trisaccharide	Apple juice: No increases Grape juice: Caffeoyl-tartaric acid Orange juice: Naringin, naringenin-trisaccharide, luteolin-rutinoside, quercetin-trisaccharide	Apple juice: Chlorogenic acid, phloridzin, hesperidin and total phenolic content Grape juice: No decreases Orange juice: No decreases	[59]
Calçots	US (40 kHz; 250 W for 0, 10, 25, 45 min)		Firmness was not significantly affected	No changes in total phenolic content	No increases	Total phenolic content	[60]
Tomato, lettuce, green pepper, red pepper, zucchini	US (40 kHz; 250 W for 20 min)		No information provided about structure	↑ Total phenolic content in all products	Total phenolic content in green pepper and lettuce	Total phenolic content in tomato, red pepper and zucchini	[61]
Juçara based smoothie	US (220 W for 7 min)		Microstructure similar to untreated smoothie but higher particle size (D_4,3_)	No changes in total phenolic content nor total anthocyanins	Total anthocyanins	No decreases	[62]
Carrot	PEF (five pulses of 3.5 kV/cm)		↓ Firmness Degradation of cell walls	↓ Total phenolic content, coumaroylquinic acid, caffeic acid, caffeic acid arab/xiloside, caffeoylshikimic acid, 3-, 4-, 5-caffeoylquinic acid, dicaffeoylquinic acid, caffeic acid derivative, ferulic acid glucoside, ferulic acid coumaroyl glucoside, ferulic acid caffeoyl glucoside ↑ Coumaric acid, caffeoylferuloylquinic acid, caffeic acid arabinoside glucoside, ferulic acid, 3-feruloylquinic acid	Total phenolic content, caffeoylshikimic acid, caffeoylferuloylquinic acid, isoferulic acid, ferulic acid glucoside, ferulic acid caffeoyl glucoside, quercetin-3-O-galactoside,	3-, 4-, 5-caffeoylquinic acid, caffeic acid arabinoside glucoside, caffeic acid Glu acetyl glucoside, ferulic acid, feruloylquinic acid derivative	[30]
Fruit juice-based beverage mixed with water	PEF (35 kV/cm for 1800 μs) High pressure processing (HPP) (400 MPa for 5 min)		No information provided about structure	PEF: ↑ Caffeic acid, ferulic acid ↓ Total phenolic content chlorogenic acid, p-coumaric acid, p-hydroxybenzoic acid, hesperidin, quercetin, rutin HPP: ↑ Caffeic acid ↓ Total phenolic content, p-coumaric acid, p-hydroxybenzoic acid, quercetin, rutin	PEF: Caffeic acid, p-coumaric, hesperidin, quercetin, rutin HPP: Total phenolic content, caffeic acid, p-coumaric, hesperidin, quercetin, rutin	PEF: Total phenolic content, chlorogenic acid, ferulic acid, p-hydroxybenzoic acid HPP: Ferulic acid	[63]
Fruit juice-based beverage mixed with milk	PEF (35 kV/cm for 1800 μs) HPP (400 MPa for 5 min)		No information provided about structure	PEF and HPP: ↑ Total phenolic content, caffeic acid, chlorogenic acid, p-coumaric acid, p-hydroxybenzoic acid, hesperidin, naringenin, quercetin ↓ ferulic acid, rutin	PEF and HPP: Total phenolic content, caffeic acid, chlorogenic acid, ferulic acid, p-coumaric acid, p-hydroxybenzoic acid, hesperidin, quercetin, rutin	No decreases	[63]
Fruit juice-based beverage mixed with soymilk	PEF (35 kV/cm for 1800 μs) HPP (400 MPa for 5 min)		No information provided about structure	PEF and HPP: ↑ Total phenolic content, caffeic acid, chlorogenic acid, p-coumaric, p-hydroxybenzoic acid, hesperidin, naringenin, quercetin, rutin ↓ ferulic acid	PEF: Total phenolic content, quercetin, rutin HPP: Total phenolic content, p-hydroxybenzoic acid, hesperidin, naringenin, rutin	PEF and HPP: p-coumaric acid	[63]
Fruit juice-based beverage mixed with soymilk	PEF (35 kV/cm for 1800 μs) HPP (400 MPa for 5 min)		No information provided about structure	PEF: ↑ Total isoflavones, daidzin, genistin, glycitin, daidzein, genistein, HPP: ↑ Total isoflavones, daidzin, genistin, glycitin, daidzein, genistein, glycitein	PEF: Total isoflavones, daidzin, genistin, daidzein, genistein, HPP: Total isoflavones, daidzin, genistin, daidzein, genistein, glycitein	No decreases	[64]
Pomelo and kiwi juices	HPH (250 MPa for 10 min)		No information provided about structure	Pomelo juice: ↑ Total phenolic content ↑ Naringenin-rutinoside, isorhamnetin-rutinoside, naringenin-rutinoside-glucoside, proanthocyanidin, proanthocyanidin-glucoside. Kiwi juice: ↑ quinic acid, chlorogenic acid, caffeoyl glucoside, EGC. ↓ sinensetin	No increases	Pomelo juice: Naringenin-rutinoside, isorhamnetin-rutinoside, feruloyl-glucoside, total phenolic content Kiwi juice: Quinic acid	[65]
Apple	PEF and storage for 0 h and 24 h	0.01 kJ/kg	Unaltered toughness	0 h: ↓ 5-caffeoylquinic acid 24 h: ↑ 5-caffeoylquinic acid, total phenolic content	No increases	0 h: 5-caffeoylquinic acid Total phenolic content	[58]
		1.8 kJ/kg	↓ Toughness	0 h and 24 h: ↓ 5-caffeoylquinic acid, 4-caffeoylquinic acid, p-coumaroylquinic acid, phloretin xyloglucoside, total phenolic content 24 h: ↓ Epicatechin	0 h and 24 h: Phloretin xyloglucoside 24 h: Total phenolic content, epicatechin 5-caffeoylquinic acid, phloretin glycoside	24 h: Quercetin glycoside, quercetin xyloside, quercetin galactoside, quercetin arabinoside	
		7.3 kJ/kg	↓ Toughness	0 h and 24 h: ↓ 5-caffeoylquinic acid, 4-caffeoylquinic acid, p-coumaroylquinic acid, phloretin glucoside, phloretin xyloglucoside, total phenolic content 0 h: ↓ Epicatechin	24 h: Total phenolic content	0 h: 4-caffeoylquinic acid	
Açai juice	US (0.9, 1.8, 2.7 and 3.6 kJ/cm^3^)		No information provided about structure	↑ Total anthocyanins (3.6 kJ/cm^3^)	Total anthocyanins	No decreases	[46]
Orange juice	HPH (150 MPa reaching 68 °C for 15 s)		No information provided about structure	↑ Vicenin-2 ↓ Total flavanones, total flavonoids, apigenin-d, hesperidin	No increases	No decreases	[49]
Mandarin juices	HPH (150 MPa reaching 68 °C for 15 s)		Cell rupture and ↓ particle size	No changes	Apigenin	No decreases	[34]

PEF: Pulsed electric fields; US: Ultrasounds; HPP: High pressure processing; HPH: High pressure homogenization.

## References

[1] Rodríguez-Roque M.J., Rojas-Graü M.A., Elez-Martínez P., Martín-Belloso O. (2014). *In vitro* bioaccessibility of health-related compounds from a blended fruit juice-soymilk beverage: Influence of the food matrix. J. Funct. Foods.

[2] Minekus M., Alminger M., Alvito P., Ballance S., Bohn T., Bourlieu C., Carrì F., Boutrou R., Corredig F.M., Dupont D. (2014). A standardised static *in vitro* digestion method suitable for food—An international consensus. Food Funct. Food Funct.

[3] Ribas-Agustí A., Martín-Belloso O., Soliva-Fortuny R., Elez-Martínez P. (2018). Food processing strategies to enhance phenolic compounds bioaccessibility and bioavailability in plant-based foods. Crit. Rev. Food Sci. Nutr..

[4] Barba F.J., Mariutti L.R.B., Bragagnolo N., Mercadante A.Z., Barbosa-Cánovas G.V., Orlien V. (2017). Bioaccessibility of bioactive compounds from fruits and vegetables after thermal and nonthermal processing. Trends Food Sci. Technol..

[5] Failla M.L., Chitchumroonchokchai C., Ishida B.K. (2008). In vitro micellarization and intestinal cell uptake of cis isomers of lycopene exceed those of all-trans lycopene. J. Nutr..

[6] Nagarajan J., Ramanan R.N., Raghunandan M.E., Galanakis C.M., Krishnamurthy N.P. (2017). Carotenoids.

[7] Failla M.L., Chitchumroonchokchai C. (2005). In vitro models as tools for screening the relative bioavailabilities of provitamin A carotenoids in foods. Harvest Plus Tech. Monogr..

[8] Granado-Lorencio F., Olmedilla-Alonso B., Herrero-Barbudo C., Pérez-Sacristán B., Blanco-Navarro I., Blázquez-García S. (2007). Comparative *in vitro* bioaccessibility of carotenoids from relevant contributors to carotenoid intake. J. Agric. Food Chem..

[9] Sy C., Gleize B., Dangles O., Landrier J.-F., Veyrat C.C., Borel P. (2012). Effects of physicochemical properties of carotenoids on their bioaccessibility, intestinal cell uptake, and blood. Mol. Nutr. Food Res..

[10] Tyssandier V., Lyan B., Borel P. (2001). Main factors governing the transfer of carotenoids from emulsion lipid droplets to micelles. Biochim. Biophys. Acta.

[11] Mapelli-Brahm P., Corte-Real J., Meléndez-Martínez A.J., Bohn T. (2017). Bioaccessibility of phytoene and phytofluene is superior to other carotenoids from selected fruit and vegetable juices. Food Chem..

[12] Schweiggert R.M., Mezger D., Schimpf F., Steingass C.B., Carle R. (2012). Influence of chromoplast morphology on carotenoid bioaccessibility of carrot, mango, papaya, and tomato. Food Chem..

[13] Panozzo A., Lemmens L., Van Loey A., Manzocco L., Nicoli M.C., Hendrickx M. (2013). Microstructure and bioaccessibility of different carotenoid species as affected by high pressure homogenisation: A case study on differently coloured tomatoes. Food Chem..

[14] Palmero P., Lemmens L., Ribas-Agustí A., Sosa C., Met K., De Dieu Umutoni J., Hendrickx M., Van Loey A. (2013). Novel targeted approach to better understand how natural structural barriers govern carotenoid *in vitro* bioaccessibility in vegetable-based systems. Food Chem..

[15] Jeffery J.L., Turner N.D., King S.R. (2012). Carotenoid bioaccessibility from nine raw carotenoid-storing fruits and vegetables using an *in vitro* model. J. Sci. Food Agric..

[16] Jeffery J., Holzenburg A., King S. (2012). Physical barriers to carotenoid bioaccessibility. Ultrastructure survey of chromoplast and cell wall morphology in nine carotenoid-containing fruits and vegetables. J. Sci. Food Agric..

[17] Palafox-Carlos H., Ayala-Zavala J.F., González-Aguilar G.A. (2011). The role of dietary fiber in the bioaccessibility and bioavailability of fruit and vegetable antioxidants. J. Food Sci..

[18] Cilla A., Bosch L., Barberá R., Alegría A. (2018). Effect of processing on the bioaccessibility of bioactive compounds—A review focusing on carotenoids, minerals, ascorbic acid, tocopherols and polyphenols. J. Food Compos. Anal..

[19] Bohn T. (2014). Dietary factors affecting polyphenol bioavailability. Nutr. Rev..

[20] Jakobek L. (2015). Interactions of polyphenols with carbohydrates, lipids and proteins. Food Chem..

[21] Jakobek L., Mati P. (2019). Non-covalent dietary fiber—Polyphenol interactions and their influence on polyphenol bioaccessibility. Trends Food Sci. Technol..

[22] Oliveira A., Amaro A.L., Pintado M. (2018). Impact of food matrix components on nutritional and functional properties of fruit-based products. Curr. Opin. Food Sci..

[23] Thakur N., Raigond P., Singh Y., Mishra T., Singh B., Lal M.K., Dutt S. (2020). Recent updates on bioaccessibility of phytonutrients. Trends Food Sci. Technol..

[24] Drago S.R. (2017). Minerals. Nutraceutical and Functional Food Components.

[25] Briones-Labarca V., Venegas-Cubillos G., Ortiz-Portilla S., Chacana-Ojeda M., Maureira H. (2011). Effects of high hydrostatic pressure (HHP) on bioaccessibility, as well as antioxidant activity, mineral and starch contents in Granny Smith apple. Food Chem..

[26] Cilla A., Barberá R., López-García G., Blanco-Morales V., Alegría A., Garcia-Llatas G. (2019). Impact of Processing on Mineral Bioaccessibility/Bioavailability.

[27] Khaneghah A.M., Bagher Hashemi S.M., Es I., Gholamhosseinpour A., Loizzo M.R., Giardinieri A., Pacetti D., Pourmohammadi K., Ferreira D.S. (2019). Water-Soluble Vitamins.

[28] Gironés-Vilaplana A., Villaño D., Marhuenda J., Moreno D.A., García-Viguera C. (2017). Vitamins. Nutraceutical and Functional Food Components.

[29] Leong S.Y., Du D., Oey I. (2018). Pulsed Electric Fields enhances calcium infusion for improving the hardness of blanched carrots. Innov. Food Sci. Emerg. Technol..

[30] López-Gámez G., Elez-Martínez P., Quiles-Chuliá A., Martín-Belloso O., Hernando-Hernando I., Soliva-Fortuny R. (2021). Effect of pulsed electric fields on carotenoid and phenolic bioaccessibility and their relationship with carrot structure. Food Funct..

[31] González-Casado S., Martín-Belloso O., Elez-Martínez P., Soliva-Fortuny R. (2018). Application of pulsed electric fields to tomato fruit for enhancing the bioaccessibility of carotenoids in derived products. Food Funct..

[32] Bot F., Verkerk R., Mastwijk H., Anese M., Fogliano V., Capuano E. (2018). The effect of pulsed electric fields on carotenoids bioaccessibility: The role of tomato matrix. Food Chem..

[33] Jayathunge K.G.L.R., Stratakos A.C., Cregenzán-Albertia O., Grant I.R., Lyng J., Koidis A. (2017). Enhancing the lycopene *in vitro* bioaccessibility of tomato juice synergistically applying thermal and non-thermal processing technologies. Food Chem..

[34] Sentandreu E., Stinco C.M., Vicario I.M., Mapelli-Brahm P., Navarro J.L., Meléndez-Martínez A.J. (2020). High-pressure homogenization as compared to pasteurization as a sustainable approach to obtain mandarin juices with improved bioaccessibility of carotenoids and flavonoids. J. Clean. Prod..

[35] Zhang W., Yu Y., Xie F., Gu X., Wu J., Wang Z. (2019). High pressure homogenization versus ultrasound treatment of tomato juice: Effects on stability and in vitro bioaccessibility of carotenoids. Lwt.

[36] Anese M., Bot F., Panozzo A., Mirolo G., Lippe G. (2015). Effect of ultrasound treatment, oil addition and storage time on lycopene stability and *in vitro* bioaccessibility of tomato pulp. Food Chem..

[37] Anese M., Mirolo G., Beraldo P., Lippe G. (2013). Effect of ultrasound treatments of tomato pulp on microstructure and lycopene *in vitro* bioaccessibility. Food Chem..

[38] Mercado-Mercado G., Montalvo-González E., González-Aguilar G.A., Alvarez-Parrilla E., Sáyago-Ayerdi S.G. (2018). Ultrasound-assisted extraction of carotenoids from mango (*Mangifera indica* L. ‘Ataulfo’) by-products on in vitro bioaccessibility. Food Biosci..

[39] Cano M.P., Gómez-Maqueo A., Fernández-López R., Welti-Chanes J., García-Cayuela T. (2019). Impact of high hydrostatic pressure and thermal treatment on the stability and bioaccessibility of carotenoid and carotenoid esters in astringent persimmon (*Diospyros kaki* Thunb, var. Rojo Brillante). Food Res. Int..

[40] Moelants K.R.N., Lemmens L., Vandebroeck M., Van Buggenhout S., Van Loey A.M., Hendrickx M.E. (2012). Relation between particle size and carotenoid bioaccessibility in carrot- and tomato-derived suspensions. J. Agric. Food Chem..

[41] Knockaert G., Pulissery S.K., Colle I., Van Buggenhout S., Hendrickx M., Loey A. (2012). Van Lycopene degradation, isomerization and *in vitro* bioaccessibility in high pressure homogenized tomato puree containing oil: Effect of additional thermal and high pressure processing. Food Chem..

[42] Buniowska M., Carbonell-Capella J.M., Frigola A., Esteve M.J. (2017). Bioaccessibility of bioactive compounds after non-thermal processing of an exotic fruit juice blend sweetened with *Stevia rebaudiana*. Food Chem..

[43] Colle I., Van Buggenhout S., Van Loey A., Hendrickx M. (2010). High pressure homogenization followed by thermal processing of tomato pulp: Influence on microstructure and lycopene *in vitro* bioaccessibility. Food Res. Int..

[44] Knockaert G., Lemmens L., Van Buggenhout S., Hendrickx M., Van Loey A. (2012). Changes in β-carotene bioaccessibility and concentration during processing of carrot puree. Food Chem..

[45] Liu X., Liu J., Bi J., Yi J., Peng J., Ning C., Wellala C.K.D., Zhang B. (2019). Effects of high pressure homogenization on pectin structural characteristics and carotenoid bioaccessibility of carrot juice. Carbohydr. Polym..

[46] de Souza Carvalho L.M., Lemos M.C.M., Sanches E.A., da Silva L.S., de Araújo Bezerra J., Aguiar J.P.L., das Chagas do Amaral Souza F., Alves Filho E.G., Campelo P.H. (2020). Improvement of the bioaccessibility of bioactive compounds from Amazon fruits treated using high energy ultrasound. Ultrason. Sonochem..

[47] Cilla A., Alegría A., de Ancos B., Sánchez-Moreno C., Cano M.P., Plaza L., Clemente G., Lagarda M.J., Barberá R. (2012). Bioaccessibility of tocopherols, carotenoids, and ascorbic acid from milk- and soy-based fruit beverages: Influence of food matrix and processing. J. Agric. Food Chem..

[48] Zhong S., Vendrell-Pacheco M., Heskitt B., Chitchumroonchokchai C., Failla M., Sastry S.K., Francis D.M., Martin-Belloso O., Elez-Martínez P., Kopec R.E. (2019). Novel Processing Technologies as Compared to Thermal Treatment on the Bioaccessibility and Caco-2 Cell Uptake of Carotenoids from Tomato and Kale-Based Juices. J. Agric. Food Chem..

[49] Stinco C.M., Sentandreu E., Mapelli-Brahm P., Navarro J.L., Vicario I.M., Meléndez-Martínez A.J. (2020). Influence of high pressure homogenization and pasteurization on the *in vitro* bioaccessibility of carotenoids and flavonoids in orange juice. Food Chem..

[50] Meléndez-Martínez A.J., Paulino M., Stinco C.M., Mapelli-Brahm P., Wang X.D. (2014). Study of the time-course of cis/trans (Z/E) isomerization of lycopene, phytoene, and phytofluene from tomato. J. Agric. Food Chem..

[51] Cervantes-Paz B., de Jesús Ornelas Paz J., Pérez-Martínez J.D., Reyes-Hernández J., Zamudio-flores P.B., Rios-velasco C., Ibarra-Junquera V., Ruiz-Cruz S. (2016). Effect of pectin concentration and properties on digestive events involved on micellarization of free and esterified carotenoids. Food Hydrocoll..

[52] Gence L., Servent A., Poucheret P., Hiol A., Dhuique-Mayer C. (2018). Pectin structure and particle size modify carotenoid bioaccessibility and uptake by Caco-2 cells in citrus juices: Vs. concentrates. Food Funct..

[53] Lemmens L., Van Buggenhout S., Van Loey A.M., Hendrickx M.E. (2010). Particle size reduction leading to cell wall rupture is more important for the β-carotene bioaccessibility of raw compared to thermally processed carrots. J. Agric. Food Chem..

[54] Faulks R.M., Southon S. (2005). Challenges to understanding and measuring carotenoid bioavailability. Biochim. Biophys. Acta.

[55] Perez O.E., Pilosof A.M.R. (2004). Pulsed electric fields effects on the molecular structure and gelation of β -lactoglobulin concentrate and egg white. Food Res. Int..

[56] Palmero P., Lemmens L., Hendrickx M., Loey A. (2014). Van Role of carotenoid type on the effect of thermal processing on bioaccessibility. Food Chem..

[57] Huo T., Ferruzzi M.G., Schwartz S.J., Failla M.L. (2007). Impact of fatty acyl composition and quantity of triglycerides on bioaccessibility of dietary carotenoids. J. Agric. Food Chem..

[58] Ribas-Agustí A., Martín-Belloso O., Soliva-Fortuny R., Elez-Martínez P. (2019). Influence of pulsed electric fields processing on the bioaccessible and non-bioaccessible fractions of apple phenolic compounds. J. Funct. Foods.

[59] He Z., Tao Y., Zeng M., Zhang S., Tao G., Qin F., Chen J. (2016). High pressure homogenization processing, thermal treatment and milk matrix affect *in vitro* bioaccessibility of phenolics in apple, grape and orange juice to different extents. Food Chem..

[60] Zudaire L., Lafarga T., Viñas I., Abadias M., Brunton N., Aguiló-Aguayo I. (2019). Effect of Ultrasound Pre-Treatment on the Physical, Microbiological, and Antioxidant Properties of Calçots. Food Bioprocess Technol..

[61] Lafarga T., Rodríguez-Roque M.J., Bobo G., Villaró S., Aguiló-Aguayo I. (2019). Effect of ultrasound processing on the bioaccessibility of phenolic compounds and antioxidant capacity of selected vegetables. Food Sci. Biotechnol..

[62] de Oliveira Ribeiro L., Braga Pinheiro A.C., Santa Brígida A.I., Asenova Genisheva Z., Martins de Oliveira Soares Vicente A.A., Couto Teixeira J.A., Martins de Matta V., Pereira Freitas S. (2019). *In vitro* gastrointestinal evaluation of a juçara-based smoothie: Effect of processing on phenolic compounds bioaccessibility. J. Food Sci. Technol..

[63] Rodríguez-Roque M.J., Ancos B., Sánchez-Moreno C., Cano M.P., Elez-Martínez P., Martín-Belloso O. (2015). Impact of food matrix and processing on the *in vitro* bioaccessibility of vitamin C, phenolic compounds, and hydrophilic antioxidant activity from fruit juice-based beverages. J. Funct. Foods.

[64] Rodríguez-Roque M.J., De Ancos B., Sánchez-Vega R., Sánchez-Moreno C., Elez-Martínez P., Martín-Belloso O. (2020). *In vitro* bioaccessibility of isoflavones from a soymilk-based beverage as affected by thermal and non-thermal processing. Innov. Food Sci. Emerg. Technol..

[65] Quan W., Tao Y., Qie X., Zeng M., Qin F., Chen J., He Z. (2020). Effects of high-pressure homogenization, thermal processing, and milk matrix on the *in vitro* bioaccessibility of phenolic compounds in pomelo and kiwi juices. J. Funct. Foods.

[66] Alminger M., Aura A.M., Bohn T., Dufour C., El S.N., Gomes A., Karakaya S., Martínez-Cuesta M.C., Mcdougall G.J., Requena T. (2014). *In vitro* models for studying secondary plant metabolite digestion and bioaccessibility. Compr. Rev. Food Sci. Food Saf..

[67] González-Aguilar G.A., Blancas-Benítez F.J., Sáyago-Ayerdi S.G. (2017). Polyphenols associated with dietary fibers in plant foods: Molecular interactions and bioaccessibility. Curr. Opin. Food Sci..

[68] Mercado-Mercado G., Blancas-Benitez F.J., Velderrain-Rodríguez G.R., Montalvo-González E., González-Aguilar G.A., Alvarez-Parrilla E., Sáyago-Ayerdi S.G. (2015). Bioaccessibility of polyphenols released and associated to dietary fibre in calyces and decoction residues of Roselle (*Hibiscus sabdariffa* L.). J. Funct. Foods.

[69] Xia Q., Wang L., Xu C., Mei J., Li Y. (2016). Effects of germination and high hydrostatic pressure processing on mineral elements, amino acids and antioxidants in vitro bioaccessibility, as well as starch digestibility in brown rice (*Oryza sativa* L.). Food Chem..

[70] Briones-Labarca V., Muñoz C., Maureira H. (2011). Effect of high hydrostatic pressure on antioxidant capacity, mineral and starch bioaccessibility of a non conventional food: *Prosopis chilensis* seed. FRIN.

[71] Cilla A., Lagarda M.J., Alegría A., De Ancos B., Cano M.P., Sánchez-moreno C., Plaza L., Barberá R. (2011). Effect of processing and food matrix on calcium and phosphorous bioavailability from milk-based fruit beverages in Caco-2 cells. FRIN.

[72] Sánchez-Moreno C., Cano P., de Ancos B., Plaza L., Olmedilla B., Granado F., Elez-Martínez P., Martín-Belloso O., Martín A. (2004). Pulsed electric fields – processed orange juice consumption increases plasma vitamin C and decreases F2-isoprostanes in healthy humans. J. Nutr. Biochem..

[73] Sánchez-Moreno C., Cano M.P., de Ancos B., Plaza L., Olmedilla B., Granado F., Elez-Martínez P., Martín-Belloso O., Martín A. (2005). Intake of Mediterranean vegetable soup treated by pulsed electric fields affects plasma vitamin C and antioxidant biomarkers in humans. Int. J. Food Sci. Nutr..

[74] Fonteles T.V., Leite A.K.F., Silva A.R.A., Carneiro A.P.G., Miguel E.D.C., Cavada B.S., Fernandes F.A.N., Rodrigues S. (2016). Ultrasound processing to enhance drying of cashew apple bagasse puree: Influence on antioxidant properties and *in vitro* bioaccessibility of bioactive compounds. Ultrason. Sonochem..

